# Modelling the impact of MAUP on environmental drivers for *Schistosoma japonicum* prevalence

**DOI:** 10.1186/s13071-020-3987-5

**Published:** 2020-03-02

**Authors:** Andrea L. Araujo Navas, Frank Osei, Ricardo J. Soares Magalhães, Lydia R. Leonardo, Alfred Stein

**Affiliations:** 1 Faculty of Geo-information Science and Earth Observation (ITC), University of Twente, PO Box 217, 7500 AE Enschede, The Netherlands; 2 UQ Spatial Epidemiology Laboratory, School of Veterinary Science, The University of Queensland, Gatton, QLD 4343 Australia; 3 Child Health and Environment Program, Child Health Research Centre, The University of Queensland, South Brisbane, QLD 4101 Australia; 4 Department of Parasitology, College of Public Health, University of the Philippines Manila, 1000 Manila, Philippines

**Keywords:** Schistosomiasis modelling, Modifiable areal unit problem, Uncertainty, Bayesian statistics, Convolution model

## Abstract

**Background:**

The modifiable areal unit problem (MAUP) arises when the support size of a spatial variable affects the relationship between prevalence and environmental risk factors. Its effect on schistosomiasis modelling studies could lead to unreliable parameter estimates. The present research aims to quantify MAUP effects on environmental drivers of *Schistosoma japonicum* infection by (i) bringing all covariates to the same spatial support, (ii) estimating individual-level regression parameters at 30 m, 90 m, 250 m, 500 m and 1 km spatial supports, and (iii) quantifying the differences between parameter estimates using five models.

**Methods:**

We modelled the prevalence of *Schistosoma japonicum* using sub-provinces health outcome data and pixel-level environmental data. We estimated and compared regression coefficients from convolution models using Bayesian statistics.

**Results:**

Increasing the spatial support to 500 m gradually increased the parameter estimates and their associated uncertainties. Abrupt changes in the parameter estimates occur at 1 km spatial support, resulting in loss of significance of almost all the covariates. No significant differences were found between the predicted values and their uncertainties from the five models. We provide suggestions to define an appropriate spatial data structure for modelling that gives more reliable parameter estimates and a clear relationship between risk factors and the disease.

**Conclusions:**

Inclusion of quantified MAUP effects was important in this study on schistosomiasis. This will support helminth control programmes by providing reliable parameter estimates at the same spatial support and suggesting the use of an adequate spatial data structure, to generate reliable maps that could guide efficient mass drug administration campaigns.
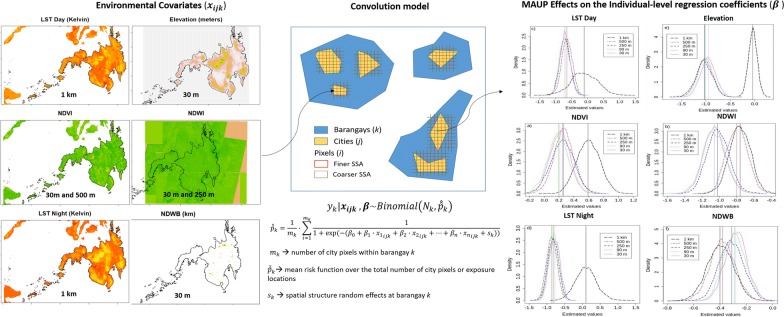

## Background

Schistosomiasis (SCH) is a water-borne neglected tropical disease of public health significance [1] associated with important morbidity outcomes in school-aged children such as malnutrition, anaemia and stunted growth in school-aged children [2, 3]. Infection is caused by skin penetration of the cercariae, the larval infective stage of the parasite, also known as schistosome. Three schistosome species cause the infection: *Schistosoma japonicum*, *S. mansoni* and *S. haematobium.* Due to its zoonotic life-cycle [4], *S. japonicum* is the hardest to control; its infection life-cycle includes the amphibious snail *Oncomelania hupensis* as the intermediate host, and humans and other mammalians as definite hosts [5, 6]. SCH affects more than 252 million people worldwide [7] especially populations living in poor conditions, where access to clean water and sanitation is limited.

Traditionally, SCH is controlled by the use of anthelminthic drugs in at-risk populations [8]. Mass drug administration campaigns identify at-risk populations by using SCH risk mapping. SCH mapping uses geographical information systems (GIS), global positioning systems and remotely sensed environmental data [9, 10]. Modelling those infections using various statistical methods have enabled the study of the distribution of populations at-risk [9, 10], and the role of the environmental variation on the geographical heterogeneity of infection burden (i.e. prevalence or intensity of infection) [11]. Statistical modelling of SCH quantifies empirical relationships between indirect morbidity indicators of public health significance and environmental risk factors. Those could be extracted from Earth Observation (EO) data such as monitor sites or satellite imagery. In addition, EO data help to interpolate the level of infection towards unsampled locations [12–14].

The robustness of SCH geographical modelling efforts is affected by uncertainties propagated from the use of EO data at various spatial and temporal scales of analysis [15]. EO data are generally constrained by their spatial and temporal scale of sampling [16]. In this study, we focus on spatial scale. Scale is a major concern in spatial epidemiology [17, 18] since it determines the significance of the various environmental risk factors on the disease distribution [19]. Spatial scale encompasses the spatial support and the spatial extent of analysis [20]. The spatial support refers to the area that each individual observation occupies in space. In the case of a raster grid, the spatial support is the spatial resolution (e.g. a 30 × 30 m-resolution Landsat pixel). The spatial extent is the spatial coverage of a set of observations (e.g. administrative units) and is gathered following a sampling scheme [20]. For a given extent, the support size and shape of spatial units may affect the patterns identified in the survey and environmental data [21, 22] and the relationship between the disease morbidity indicators and the environmental risk factors. This is known as the modifiable areal unit problem (MAUP) [23, 24]. The MAUP arises because spatial units of analysis are often created using different *ad hoc* shapes and sizes. Statistical analyses of data performed according to these varying spatial units may lead to different results (e.g. correlation and regression coefficients) [24].

Various studies investigated the consequences of ignoring MAUP effects in spatial epidemiological modelling. For instance, Hellsten et al. [25] studied the influence of using aggregated covariate data to model ammonia emissions at the farm level. They showed that the size and shape of spatial aggregation areas strongly affect the location of the emissions estimated by the model, e.g. too small areas resulting in false emission “hot spots”. Schur et al. [21] and Schur et al. [22] aggregated SCH prevalence maps to estimate endemicity for various administrative units [26]. Such aggregation showed different patterns of endemicity and intervention approaches. As a consequence, localized areas of high endemicity may not be addressed properly. In a recent study [27], we quantified the effect of pure specification bias, that originates when using group-level (i.e. aggregated) survey data at an administrative level for individual-level inferences. Equation 1 shows the common method used to model schistosomiasis. Data on the human *S. japonicum* infection variable $y$ are commonly aggregated at barangay $k$ level, $y_{k}$ has a binomial distribution with parameters $N_{k}$ and $p_{k}$ corresponding to the number of sampled individuals and the probability of infection, respectively. Parameters for this distribution are obtained from the mean of various environmental risk factors within barangay $k$ as predictors, denoted as $\bar{x}_{k}$, where γ are the barangay-level coefficients, $\gamma_{0}$ being the intercept and $\gamma_{{\left( {1 \ldots n} \right)}}$ the regression coefficients for $n$ environmental covariates (Eq. 1).

$$ y_{k} |\bar{x}_{k} ,\gamma \sim Binomial\left( {N_{k} ,\hat{p}_{k} } \right) $$

1 $$ logit(\hat{p}_{k} ) = \gamma_{0} + \gamma_{1} \cdot \bar{x}_{{2_{k} }} + \gamma_{2} \cdot \bar{x}_{{2_{k} }} + \ldots + \gamma_{n} \cdot \bar{x}_{{n_{k} }} $$

We calculated individual-level regression parameters by modifying Equation 1 into a convolution model. We observed differences ranging from − 0.19 to 0.28 between individual (i.e. γ coefficients) and group level parameter estimates and their uncertainties. High differences were observed for NDWI (0.28), LSTN (− 0.19) and LSTD (0.16). Although some covariates exhibited a less significant effect on schistosomiasis, uncertainties in their individual level coefficients were lower than the group-level regression coefficients (e.g. LSTD and elevation). We concluded that the choice of spatial support affects the model parameter estimates and their associated uncertainties by changing the within-covariates variability in exposure areas. The selection of spatial support should be further investigated as it might represent a significant source of uncertainty in SCH modelling [15].

Up to date, the majority of SCH studies have put little attention to the size of spatial support. They use EO data at various spatial supports with misaligned grids ignoring the possible consequences on the observed patterns of the data [21, 22]. Moreover, MAUP effects on the various environmental risk factors used as drivers for SCH infection have not been quantified. This is important as the relevance of the environmental risk factors on SCH depends on the scale of analysis [7, 19]. Ignoring MAUP effects might produce unreliable predictions of at-risk populations, and consequently, wrong decisions based upon inefficient mass drug administration campaigns.

The purpose of this research is to quantify MAUP effects on environmental drivers of *S. japonicum* infection. To achieve this objective we aim to: (i) aggregate and disaggregate EO data in order to bring all covariates to a the same spatial support of analysis; (ii) estimate individual-level covariate regression parameters at 30 m, 90 m, 250 m, 500 m and 1 km spatial supports, by using a convolutional model that accounts for pure specification bias; and (iii) quantify the differences between parameter estimates using five different models.

## Methods

### Study area and data on human *Schistosoma japonicum* infection

**Fig. 1:**
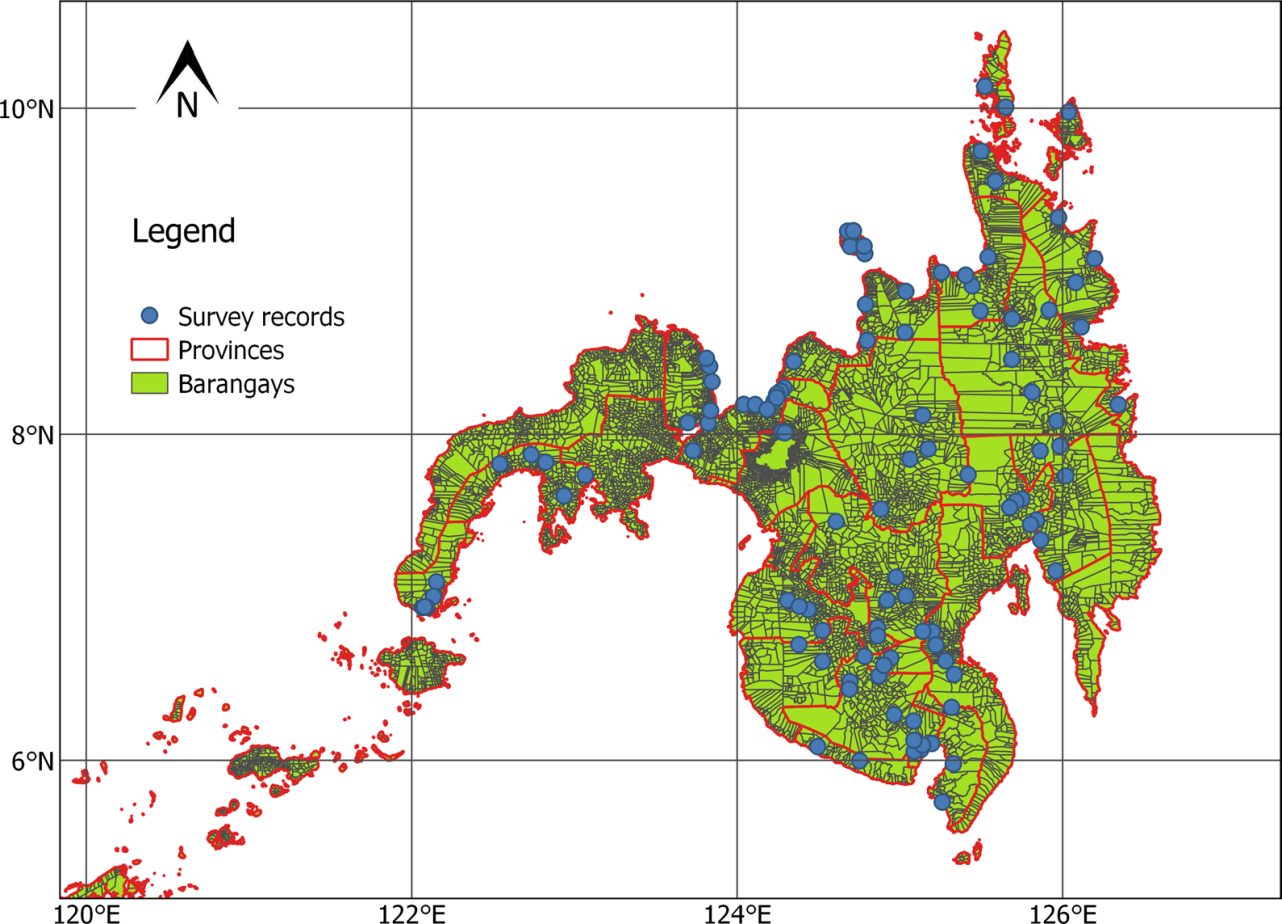
Study area: the Mindanao region in the Philippines. Blue dots are the aggregated survey data at barangay-level

We use *S. japonicum* infection data collected as part of the 2008 Nationwide Schistosomiasis Survey in the Philippines. Here, *S. japonicum* is endemic in 28 of its 81 provinces [28], with approximately 1.8 million estimated infected people [29]. The disease affects children, adolescents, and individuals with high-risk occupations, such as farmers and fishermen [29, 30]. The area of study is the region of Mindanao in the Philippines (Fig. 1). This area was selected due to the high response rate of 70.9% of the individuals to the 2008 survey [31, 32] and the good spatial coverage of the sampling.

A two-stage systematic cluster sampling was used where stratification was done using high, medium and low prevalence levels, obtained from the 1994 World Bank-assisted Philippine Health Development Programme. Provinces and sub-municipalities called barangays were the primary and secondary sampling units, respectively. A barangay is the smallest administrative division in the Philippines, numbering from 58 to 1158 within a single province. In total, 11 provinces with high (≥ 2%) and medium (0.091–1.99%) prevalence rates were included, while 9 low-prevalence (0.04–0.09%) provinces were randomly selected. Within the selected provinces, barangays with high prevalence rates were surveyed. In total, between 2 and 10 barangays were surveyed per province, resulting in 108 out of 10,021 barangays that were surveyed in Mindanao.

For *S. japonicum* diagnosis, a Kato-Katz thick smear examination [32] was used based on a two-sample stool collection. However, due to inconsistencies in the second stool sample submission, only the results of the first sample were available [8]. Samples were taken from people aged two years and above and were analysed using a microscope. Active infection was indicated by the presence of *S. japonicum* eggs.

Data such as age and gender were recorded for 19,763 individuals. Barangay and province information for each individual was recorded but not georeferenced. For this reason, individual-level survey data were aggregated and geolocated to the centroids of the 108 barangays. We used a probability of infection in barangay $k$ as our disease outcome variable. We obtained an up-to-date barangay centroids shape file from DIVA geographical information system [33]. More details about the sampling design and surveyed information can be found in Leonardo et al. [31, 34].

### Environmental risk factors

We included in our analysis six relevant environmental risk factors for SCH transmission [35, 36]. These are the nearest distance to water bodies (NDWB), the normalized difference vegetation index (NDVI), the normalized difference water index (NDWI), land surface temperature at day (LSTD) and at night (LSTN), and elevation (E). NDWB shows the accessibility of people to water bodies that represent potential infection foci as they may contain contaminated snail hosts that release the infective larval stages of the parasite [8]. NDVI is and indicator of flooded vegetation [8], particularly rice-paddy fields, and environmental moisture [37, 38]. Both are an important risk factor for Asian SCH [39]. NDWI was used as a proxy indicator of flooding [37, 40] showing potentially hidden water bodies. LSTD and LSTN are determinant for the survival of snail larval stages [41, 42] and are used as proxies for water temperature given that the thermal condition of shallow waters usually reflects the ambient temperature of the air [8]. Elevation is relevant for SCH transmission as the local topography of the area determines the presence of snails [43, 44]. For instance, at lower altitudes the risk of finding snails increases.

NDWB values range from 0.17 to 26.2 km and were calculated using the closest facility network analysis tool from ArcGIS [45]. We used the river and road network, and the cities and hamlets locations as input for the network. Rivers and roads were extracted from the Open Street Map Project in the Philippines [46]. Cities and hamlet locations were obtained from the National Mapping and Resource Information Authority from The Philippines [47] data base from 2010. We calculated the nearest distance from each city and hamlet to a water body following a road and interpolated those values within all surveyed barangays towards a spatial support of 30 m.

NDVI values range from 0 to 0.84 and were obtained from two sources of information, i.e. a series of Landsat 5 images from 2008 with a spatial support of 30 m and the MODIS MOD13Q1 product with a spatial support of 250 m. NDWI values range from 0.06 to 0.61 and were also obtained from two sources of information, i.e. a Landsat 5 imagery product from 2008 with a spatial support of 30 m and the annual composite from Landsat 7 from 2008 derived from Google Earth Engine with a spatial support of 500 m. LSTD values range from 297.77 to 309.52 °K and LSTN ranges from 289.73 to 297.29 °K. LSTD and LSTN values were derived from MODIS MOD11A2_LST product with a spatial support of 1 km. Finally, elevation values range from 0 to 969.57 m was obtained from ASTER GDEM version 2 from USGS [48] with a spatial support of 30 m. All covariates were set to a common coordinate system UTM zone 51N and were standardized to have mean = 0 and standard deviation = 1 before being used. Table 1 summarizes all sources of information.

**Table 1:** Environmental variables description

Environmental variable	Spatial resolution	Temporal resolution	Data type	Original coordinate system	Data source
Elevation	30 m	na	Raster	EPSG:4326	ASTER GDEM V2 from USGS
NDVI	250 m	2008	Raster	EPSG:4326	MOD13Q1
	30 m	2008	Raster	EPSG:4326	Landsat 5
NDWI	500 m	2008	Raster	EPSG:32651	Landsat 7, 1-year composite
	30 m	2008	Raster	EPSG:4326	Landsat 5
LST	1 km	2008	Raster	EPSG:4326	MOD11A2
NDWB	250 m	2010	Raster	EPSG:32651	Derived from closest facility network using roads, urban areas, river network and water bodies

*Abbreviations*: NDVI, normalized difference vegetation index; NDWI, normalized difference water index; LST, land surface temperature day and night; NDWB, nearest distance to water bodies. USGS, United States Geological Survey; na, not applicable

#### Modifying the areal units of analysis

From now onwards, we will refer to an area unit as the spatial support of analysis (SSA). We used five SSAs, with a spatial support equal to 30 m, 90 m, 250 m, 500 m and 1 km, respectively. These spatial supports increase when going from low to high data aggregation. These values were selected based upon the commonly used spatial supports at which the environmental information is originally provided.

For NDVI, SSA = 30 m, we obtained NDVI values from Landsat 5 images. Many of these images presented gaps due to the presence of clouds. These gaps were covered using disaggregated NDVI MODIS images at the Landsat resolution. Disaggregation was performed using a linear model that predicted NDVI Landsat values based on NDVI MODIS values. NDVI values were obtained by merging the original and predicted Landsat NDVI values. For SSA = 90 m, we aggregated the previously merged NDVI values using their mean. For SSA = 250 m, we used the NDVI MODIS product directly. Finally, for SSA = 0.5 and 1 km, we aggregated the NDVI mean values from MODIS.

NDWI values were obtained from the Landsat 5 images. Gaps in some of these images were covered using disaggregated NDWI composite images at the Landsat resolution. Disaggregation towards SSA = 30 m was done by interpolating NDWI values using ordinary kriging. For SSA = 90 m and 250 m, we aggregated the combined 30 m NDWI using its mean. For SSA = 500 m, we directly used the Landsat 7 composite. Finally, for SSA = 1 km, we aggregated the mean of the original Landsat 7 composite.

To obtain LSTD and LSTN values for SSA = 30 m, we disaggregated the original MODIS values by using ordinary kriging interpolation. For SSA = 90 m, 250 m and 500 m, we aggregated the previously interpolated values using their mean. For SSA = 1 km, we used directly LSTD and LSTN from MODIS.

The interpolated NDWB values for SSA = 30 m were used to obtain NDWB for SSA = 90 m, 250 m, 500 m and 1 km by aggregating the mean values. For elevation, we directly used the original 30 m SSA Aster images. For SSA = 90 m, 250 m, 500 m and 1 km, we aggregated the mean values of the original Aster images.

### Modelling *Schistosoma japonicum* infection under the MAUP

#### Convolution model

We modelled human *S. japonicum* infection at the five increasing SSAs using a convolution model that accounts for pure specification bias [27]. Pure specification is a source of uncertainty [11, 49] that produces loss of information on the real relationship between the disease and the environmental covariate data, when using aggregated survey data in a non-linear model, for example, for individual-level inferences [50]. It is called ‘pure’ because it specifically addresses model specification bias [51], and it biases the estimates because any direct link between exposure and health outcomes is imperfectly measured [52]. This is because the regression function does not approximate the real relationship between the affected population and their exposure [27]. Pure specification bias can be reduced as the within area exposure is more homogenous [50]. This could be done by having a finer partition of space at which environmental risk factors are available [50, 53].

**Fig. 2:**
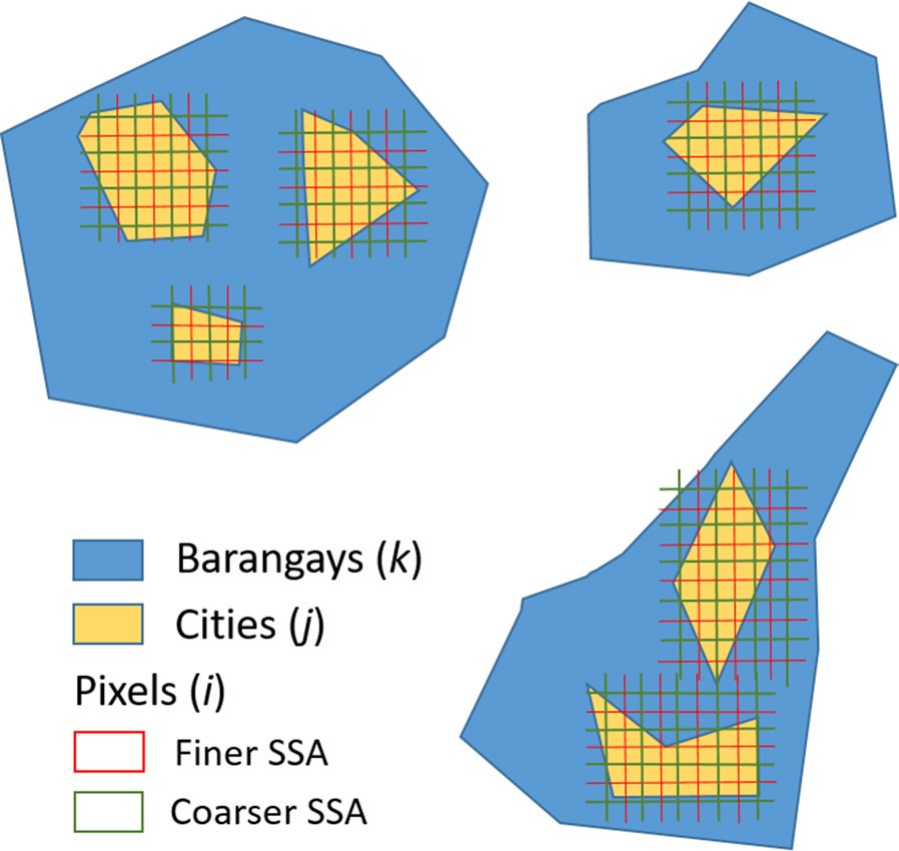
Environmental risk factors extraction at pixel-level from cities within barangays

In this study, we propose to minimize and quantify pure specification bias by extracting covariate information from cities within barangays (Fig. 2) and by modelling the disease using a convolution model [53]. The city level is the finest available extent of analysis. Cities thus serve as a proxy for individual-level exposure locations. We identified all cities within the surveyed barangays using Google Earth Images. Available cities were extracted from the 2010 build-up data base from the National Mapping and Resource Information Authority of the Philippines [54]. We completed unavailable cities using Google Earth Images.

For the convolution model, we used the aggregate data method proposed by Prentice and Sheppard [55]. For each SSA, we obtained covariate information $x$ for image pixel $i$ belonging to a city $j$ within a specific barangay $k$ (Fig. 2). Let $n = 6$be the number of covariates $x_{ijk}$ measured at locations $s_{ijk}$ $i = 1, \ldots ,m_{k}$ where $m_{k}$ denotes the number of city pixels within barangay $k$. Note that with an increasing resolution, the possibility increases that there are no pixel points falling in cities of the within-pixel sizes. Data on the human *S. japonicum* infection are available at individual-level recorded within a barangay $k$. Because the exact response locations of the individual-level data are unknown, we aggregated them to their corresponding barangay centroid, denoted by $y_{k}$. To estimate the average probability of infection of the individuals in barangay $k$ and the individual level coefficients $\beta$, we obtained the mean risk function $\hat{\hat{p}}_{k}$ over the total number of city pixels or exposure locations (Eq. 2). We accounted for the spatial variability at barangay-level by adding a spatial structure random effects term $s_{k}$. Pure specification bias results as $\gamma \ne \beta$ and is then minimized by using the individual-level regression coefficients $\beta$ instead of the group-level coefficients $\gamma$. The accompanying uncertainties are quantified by the difference between the group an individual-level credible intervals [27] for each SSA. The convolution model used is of the form:

$$ y_{k} |x_{ijk} ,\beta \sim Binomial\left( {N_{k} ,\hat{\hat{p}}_{k} } \right) $$

2 $$ \hat{\hat{p}}_{k} = \frac{1}{{m_{k} }} \cdot \mathop \sum \limits_{i = 1}^{{m_{k} }} \frac{1}{{1 + \exp \left( { - \left( {\beta_{0} + \beta_{1} \cdot x_{{1_{ijk} }} + \beta_{2} \cdot x_{{2_{ijk} }} + \ldots + \beta_{n} \cdot x_{{n_{ijk} }} + s_{k} } \right)} \right)}}. $$

#### Model implementation

Five models were implemented, all including an intercept ($\beta_{0}$), pixel-level environmental variables ($x_{ijk}$ = NDVI, NDWI, LSTD, LSTN, E, NDWB) and their corresponding individual-level coefficients $\beta$. Collinearity between covariates was assessed with the Pearsonʼs correlation coefficient. All covariates were standardized to have mean = 0 and standard deviation = 1.

The intercept $\beta_{0}$ was given a diffuse uniform prior distribution with wide bounds $\beta_{0}$ $\sim U\left[ { - 100,100} \right]$. The other $\beta$ parameters were given a diffuse normal distribution $\beta \varvec{ }\sim N\left[ {0,\frac{1}{{\sigma^{2} }}} \right]$, with $\sigma$ uniformly distributed on a wide range of $\sigma \sim U\left[ {0, 100} \right]$. These distributions avoid overestimating the parameters [56] and allow a good sequences mixing used for Markov Chain Monte Carlo (MCMC) simulations, contributing to a fast convergence [57].

Prior information for the spatially structured random effects was based upon a geostatistical model that can be used as a sampling distribution for continuous spatial data [58]. The vector of random variables $s$ associated with point locations ($x_{k} ,y_{k}$), $k = 1, \ldots ,K,$ was modelled with a multivariate normal distribution $s\sim MVN_{K} \left[ {\mu , \varSigma_{ab} } \right]$ with a mean $\mu$ = 0 and a covariance matrix $\varSigma_{ab} = \sigma^{2} \cdot \exp \left[ { - \left( {\phi \cdot d_{ab} } \right)^{\kappa } } \right]$ defined by a powered exponential spatial decaying correlation function.

The covariance matrix $\varSigma_{ab}$ is specified as a function of the distances $d_{ab}$ between barangay centroids $a$and $b$, with the rate of decline of spatial correlation per unit of distance $\phi$, the scalar parameter representing the overall variance $\sigma^{2}$ and the scalar parameter $\kappa$ controlling the amount of spatial smoothing. Because extreme values of $\kappa$ (0 and 2) could lead to undesirable smoothing, we used $\kappa = 1.$ Prior information for $\phi$ was set to be uniform:$\phi \sim U\left[ {2{\text{E}}10^{ - 7} ,3{\text{E}}10^{ - 3} } \right]$. These values give a diffuse but plausible prior range of correlations between 0.1–0.99 at the minimum distance between points (575 m) and between 0–0.3 at the maximum distance between points (< 552 km), assisting identifiability [59]. For $\sigma^{2}$, a half-normal distribution was selected: $\sigma^{2} \sim HN\left[ {0,1} \right]$ to restrict the prior $\sigma^{2}$ to positive values and avoid problems with convergence [56, 60].

To run the model, we used three sequences or chains with 50,000 iterations. This number of iterations ensured that the simulations were representative of target distributions and a stable convergence [57]. In order to diminish the influence of starting values, we discarded the first half of each sequence [57] using a ‛burn-in’ of 25,000 iterations. Convergence was monitored visually and statistically by inspecting the trace plots, and by checking the $\hat{R}$statistic [61, 62] also called the potential scale reduction factor. This potential scale reduction factor assesses sequences mixing by comparing the between and within variation. An $\hat{R}$ value < 1.1 indicates evidence that sequences have converged [61], while higher values suggest that an increase in the number of simulations may improve the inferences [57].

Survey and environmental data were structured in a rectangular format where columns are headed by the array name. Survey data and the codes in BUGS for the various SSA are provided in Additional file 1: Table S1 and Additional file 2: Text S1, respectively.

### Model validation

The five models were validated using two methods. The first method compared the data generated from the simulations of the predictive distribution to the observed data using a test statistic. A test statistic is a value derived from the sampled data and is used to perform hypothesis testing. This test statistic is the posterior predictive *P*-value (pp*P*-value) generated by calculating the proportion of the predicted values which are more extreme than the observed maximum, minimum and mean prevalence observed value. We calculated (i) the proportion of simulations of the data from the model for which the maximum prevalence across simulated barangays is greater than or equal to the maximum observed value, (ii) the proportion of simulations of the data from the model for which the minimum prevalence across simulated barangays is greater than or equal to the minimum observed value, and (iii) the proportion of simulations of the data from the model for which the mean prevalence across simulated barangays is greater than or equal to the mean observed value. If the model fits the data, the simulated values distribute closely around the observed values, thus, we expect a pp*P*-value of around 0.5. Otherwise, for a biased model, the pp*P*-value will be close to 0 or 1. Our aim was to test whether the model predicts a similar number of barangays with maximum and minimum prevalence values compared with the observed data. We generated pp*P-*values for maximum, minimum and mean prevalence values for the models at five increasing SSA using 75,000 simulations. The second method used the area under the curve (AUC) of the receiving operating characteristics (ROC). We applied a threshold of 0.5% (prevalence mean in Mindanao region) since we are interested in knowing the ability of the models to discriminate the mean prevalence level in the study area. We also examined the ability of the model to discriminate the number of positive cases, thus, we used a threshold of 1, which indicates the presence of at least one positive case. We used an AUC value of 70% to indicate acceptable predictive performance [8, 63].

### Software

Model implementation was done in the software OpenBUGS 3.2.3 [64, 65] (Medical Research Council, Cambridge, UK and Imperial College London, UK). It was downloaded for free at [66]. We called OpenBUGS from R using the package R2OpenBUGS [67]. The spatial models were coded using the GeoBUGS [59] function as an add-on module to OpenBUGS. GeoBUGS provides an interface to work with conditional autoregressive and geo-statistical models. Data pre-processing and Ordinary Kriging was performed in R [68].

## Results

### Modelling *Schistosoma japonicum* infection under the MAUP

#### Convolution model

**Fig. 3:**
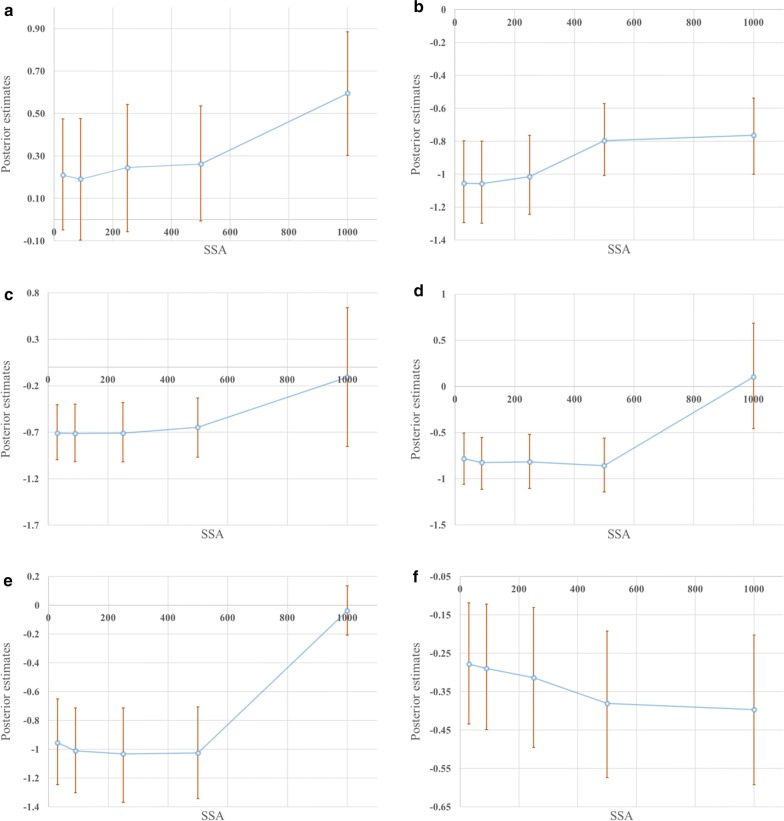
Posterior estimates and their credible intervals. **a** Normalized difference vegetation index. **b** Normalized difference water index. **c** Land surface temperature day. **d** Land surface temperature night. **e** Elevation. **f** Nearest distance to water bodies. *Abbreviation*: SSA, spatial support of analysis

**Fig. 4:**
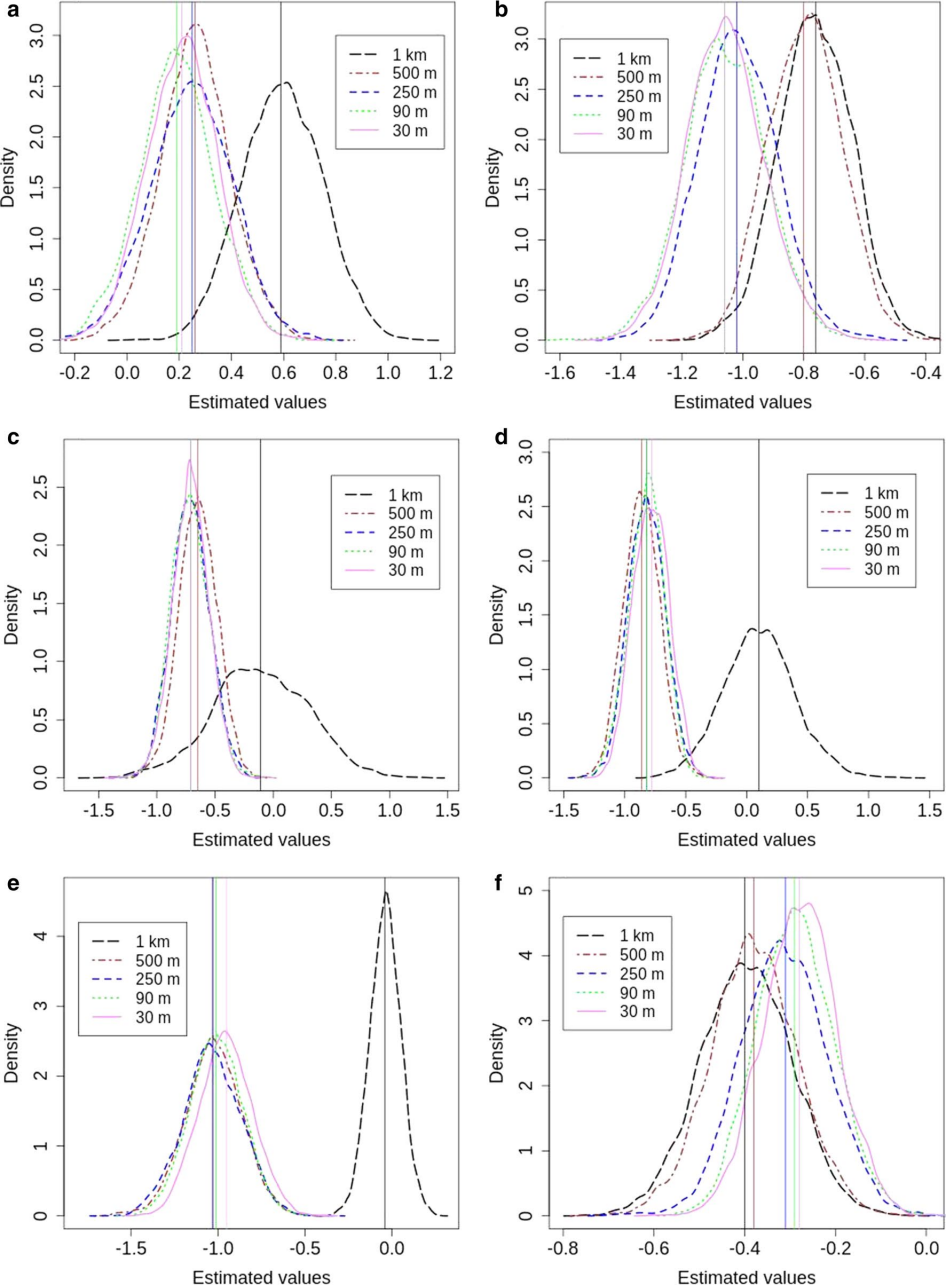
Density plots for the risk factors regression coefficients. **a** Normalized difference vegetation index. **b** Normalized difference water index. **c** Land surface temperature day. **d** Land surface temperature night. **e** Elevation. **f** Nearest distance to water bodies

Our findings show that NDVI has a non-significant effect on the prevalence of SCH infection for all SSA, except for SSA = 1 km (Additional file 3: Table S1, Fig. 3a). NDVI estimates vary gradually from 0.19 to 0.26 when increasing SSA until 500 m. For SSA = 1 km, the estimate rapidly increases to 0.59. Uncertainties are similar throughout all SSA (Fig. 4a, Table 2), slightly increasing when increasing SSA. The highest credible interval value is 0.60 for SSA = 250 m and the lowest is 0.52 for SSA = 30 m.

**Table 2:** Credible interval widths (uncertainty) at five increasing spatial supports of analysis

Spatial supports of analysis	Credible intervals width (uncertainty)					
	NDVI	NDWI	LSTD	LSTN	E	NDWB
30 m	0.52	0.50	0.59	0.56	0.59	0.32
90 m	0.57	0.50	0.62	0.56	0.59	0.33
250 m	0.60	0.48	0.64	0.59	0.66	0.36
500 m	0.54	0.44	0.64	0.58	0.64	0.38
1 km	0.58	0.46	**1.49**	**1.14**	0.34	0.39

*Note*: High uncertainty values are present in bold

*Abbreviations*: NDVI, normalized difference vegetation index; NDWI, normalized difference water index; LSTD, land surface temperature day; LSTN, land surface temperature night; NDWB, nearest distance to water bodies

NDWI has a significant negative effect on the prevalence of SCH infection throughout all SSAs (Additional file 3: Table S1, Fig. 3b). When SSA increases, parameter estimates increase from − 1.06 to − 0.76, coming somewhat closer to zero. We found similar estimates for SSA = 30 m, 90 m and 250 m (i.e. from − 1.06 to − 1.02), and for SSA = 500 m and 1 km (i.e. from − 0.8 to − 0.76) (Fig. 4b). Uncertainty values are similar for all SSAs and show a slight decrease when increasing SSA (Fig. 4b, Table 2). The highest uncertainty value equals 0.54 for SSA = 30 m, and the lowest value equals 0.44 for SSA = 500 m.

LSTD has a significant negative effect on the prevalence of SCH infection for almost all SSA, except for SSA = 1 km (Additional file 3: Table S1, Fig. 3c). Similar parameter estimates equal to − 0.71 are obtained for SSA = 30 m, 90 m and 250 m, while the parameter estimate increases slightly to − 0.65 for SSA = 500 m. For SSA = 1 km, there is a noticeable increase in the parameter estimate to − 0.01 (Figs. 3c, 4c). Uncertainty increases from 0.59 to 0.64 when increasing SSA from 30 m to 500 m, but for SSA = 1 km there is a considerable increase in uncertainty to 1.49 (Fig. 4c).

LSTN has a significant negative effect on the prevalence of SCH infection for almost all SSA, except for SSA = 1 km (Additional file 3: Table S1, Fig. 3d). Parameter estimates increase from − 0.78 to − 0.86 while increasing SSA from 30 m to 500 m. For SSA = 1 km, the parameter estimate rapidly goes up to 0.1 (Figs. 3d, 4d). Uncertainty increases slightly from 0.56 to 0.58 when increasing SSA from 30 m to 500 m, but it increases considerably to 1.14 for SSA = 1 km (Table 2, Fig. 4d).

Elevation has a significant negative effect on the prevalence of SCH infection for all SSA, except for SSA = 1 km (Additional file 3: Table S1, Fig. 3e). When increasing SSA from 30 m to 500 m, parameter estimates slightly decrease from − 0.95 to − 1.03. For SSA = 1 km, the parameter estimate considerably increases to − 0.04 (Figs. 3e, 4e). Uncertainty values vary from 0.59 to 0.64 when increasing SSA from 30 m to 500 m. For SSA = 1 km, uncertainty considerably decreases to 0.35 (Table 2, Fig. 4). The lowest uncertainty value is 0.35 for SSA = 1 km and the highest is 0.66 for SSA = 250 m.

Finally, NDWB has a significant negative effect on the prevalence of SCH infection for all SSA (Additional file 3: Table S1, Fig. 3f). We found similar parameter estimates of − 0.28, − 0.29 and − 0.31 for SSA = 30 m, 90 m and 250 m, respectively, and estimates of − 0.38 and − 0.4 for SSA = 500 m and 1 km, respectively (Fig. 3f). Uncertainties constantly increase from 0.32 to 0.39 (Table 2, Fig. 4f) when increasing SSA.

Intercept values range from − 6.02 to − 6.17 for almost all SSAs, except for SSA = 1 km, where it is equal to − 5.49. The rate of decay of spatial autocorrelation ($\varphi$) ranges from 1.65 × 10^−5^ to 2.81 × 10^−4^ for SSAs = 1 km and 500 m, respectively.

Our findings show high and moderate correlation and determination ($R^{2}$) coefficient values between the SSAs and all environmental covariates estimates (Table 3) with correlation coefficients ranging from − 0.94 to 0.94 and $R^{2}$ values from 0.6 to 0.86, respectively. Correlation coefficients between the SSAs and uncertainties are high for LSTD, LSTN and NDWB with the values of 0.91, 0.9 and 0.91, respectively. Determination coefficients $R^{2}$ between the SSAs and uncertainties in the covariate estimates are moderate for LSTD, LSTN and NDWB with the values of 0.76, 0.75 and 0.76, respectively (Table 3). Uncertainties in NDVI and NDWI estimates do not show any correlation with SSAs (Table 3).

**Table 3:** Correlation and determination coefficients between the spatial supports of analysis (SSAs) and environmental covariates estimates and uncertainties

Covariates	Estimates			Uncertainties		
	Correlation coefficient	Determination coefficient (*R*^2^)	*P*-value	Correlation coefficient	Determination coefficient (*R*^2)^	*P*-value
NDVI	0.94	0.85	0.02	0.32	− 0.2	0.6
NDWI	0.93	0.81	0.02	− 0.3	− 0.2	0.6
LSTD	0.92	0.8	0.03	0.91	0.76	0.03
LSTN	0.86	0.6	0.06	0.9	0.75	0.04
E	0.86	0.65	0.06	− 0.78	0.48	0.12
NDWB	− 0.94	0.86	0.02	0.91	0.76	0.03
Variance	0.64	0.21	0.25	− 0.54	0.06	0.35

#### Influence on predictions

**Fig. 5:**
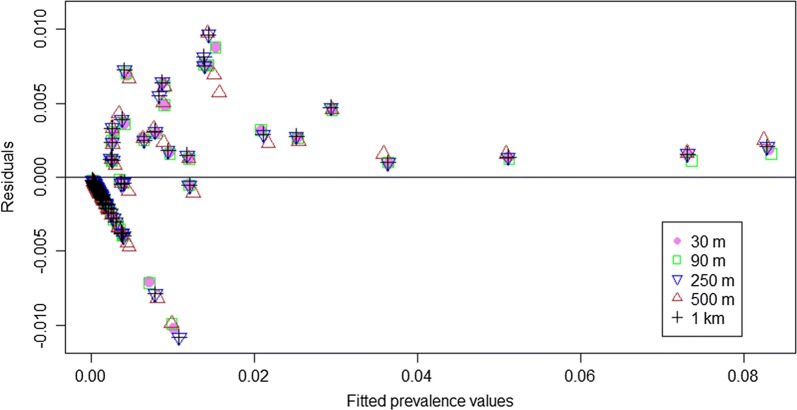
Residual plot for the five increasing spatial supports of analysis. The x-axis represents the fitted prevalence values for the five spatial supports of analysis. The y-axis represents the residuals calculated by the difference between the observed and predicted prevalence values

Differences between observed and predicted prevalence values are similar for the five SSA models (Fig. 5). Variation in these differences is highest between the 30 m and 1 km models ($$ R^{2} = 0.94 $$) and lowest between the 30 m and 90 m models ($$ R^{2} = 0.99) $$. Figure 5 shows that the maximum and minimum differences are 1.11% and 0.01%, respectively, corresponding to the 1 km SSA model. For fitted prevalence values higher than 2%, all models underestimate the prevalence of infection, while for fitted prevalence values lower than 2%, overestimation and underestimation occur for the five models (Fig. 5). A plot of the residuals against prevalence from Fig. 5 serve as a visual inspection of the fit, where we realize that it is based on positive predictive predictions.

Uncertainties on the predictions are similar for the five models (Additional file 4: Figure S1). Higher differences in uncertainty were found between the 500 m and 1 km models ($R^{2} = 0.96$), and lower differences were found between the 90 m and 250 m models ($R^{2} = 0.99$). The highest uncertainty value is 9.23% for all the models, except the 1-km model with 8.9% and the lowest uncertainty value is 0.006% for the 1-km model.

### Model validation

**Fig. 6:**
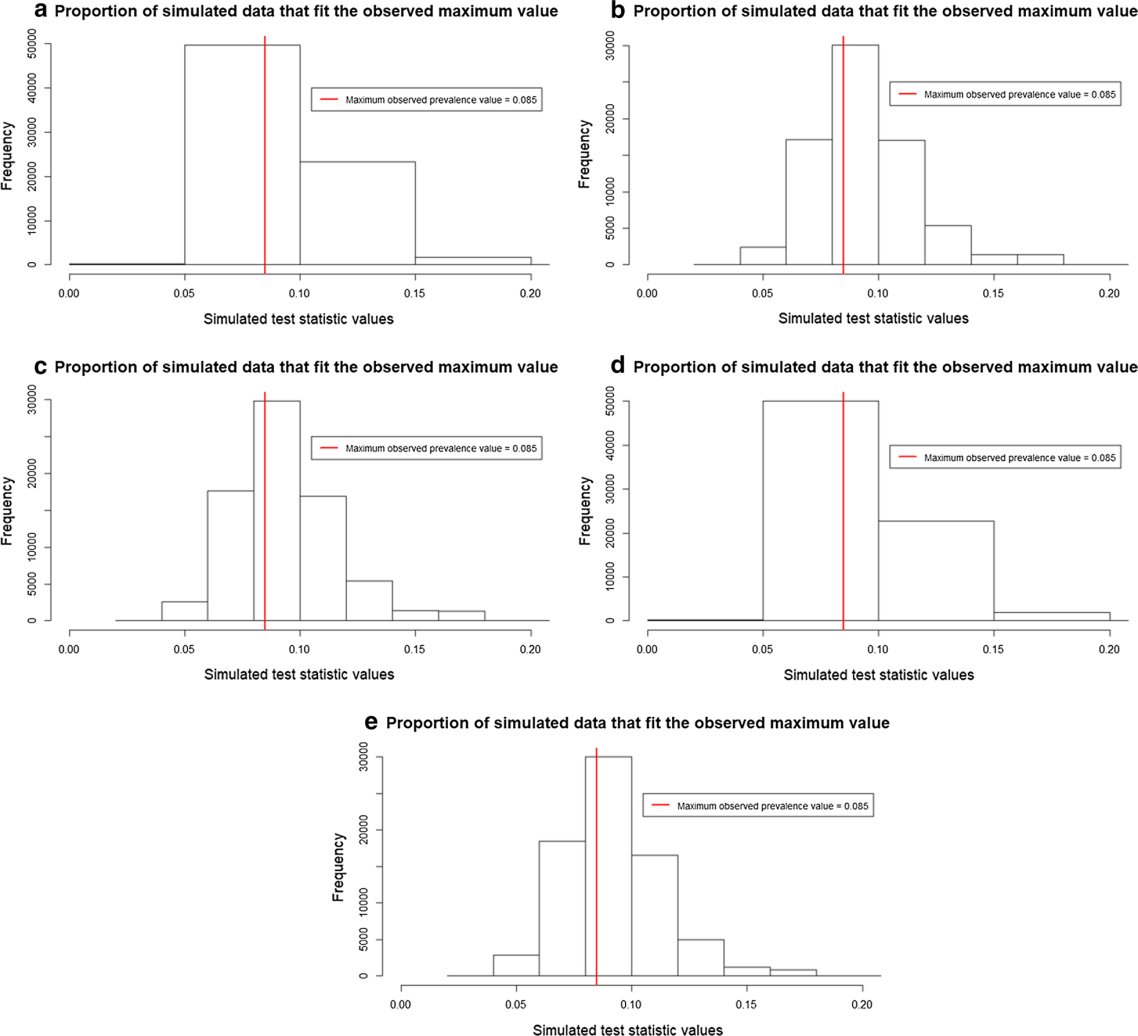
Proportion of simulated prevalence data that fit the observed maximum prevalence value. **a** SSA = 30 m. **b** SSA = 90 m. **c** SSA = 250 m. **d** SSA = 500 m. **e** SSA = 1 km. *Abbreviation*: SSA, spatial support of analysis

**Fig. 7:**
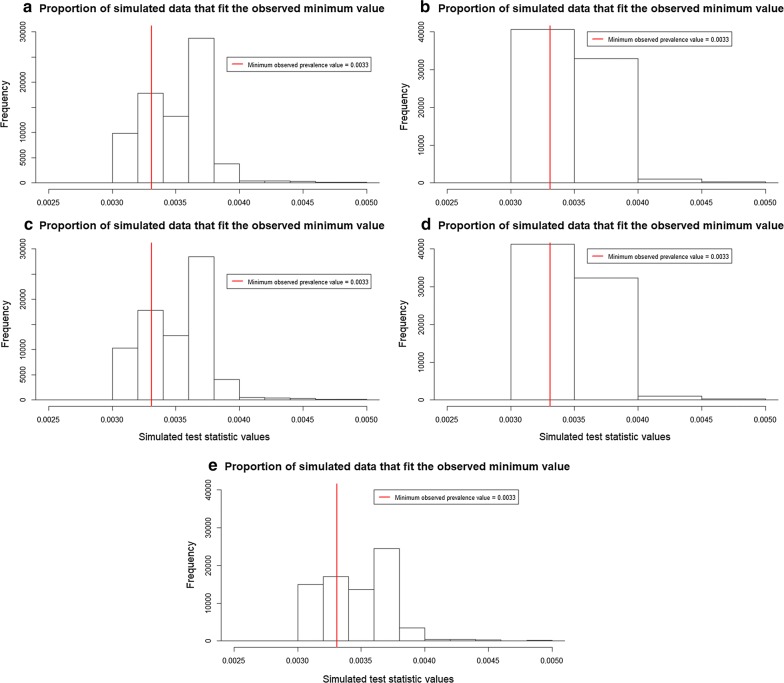
Proportion of simulated prevalence data that fit the observed minimum prevalence value. **a** SSA = 30 m. **b** SSA = 90 m. **c** SSA = 250 m. **d** SSA = 500 m. **e** SSA = 1 km. *Abbreviation*: SSA, spatial support of analysis

**Fig. 8:**
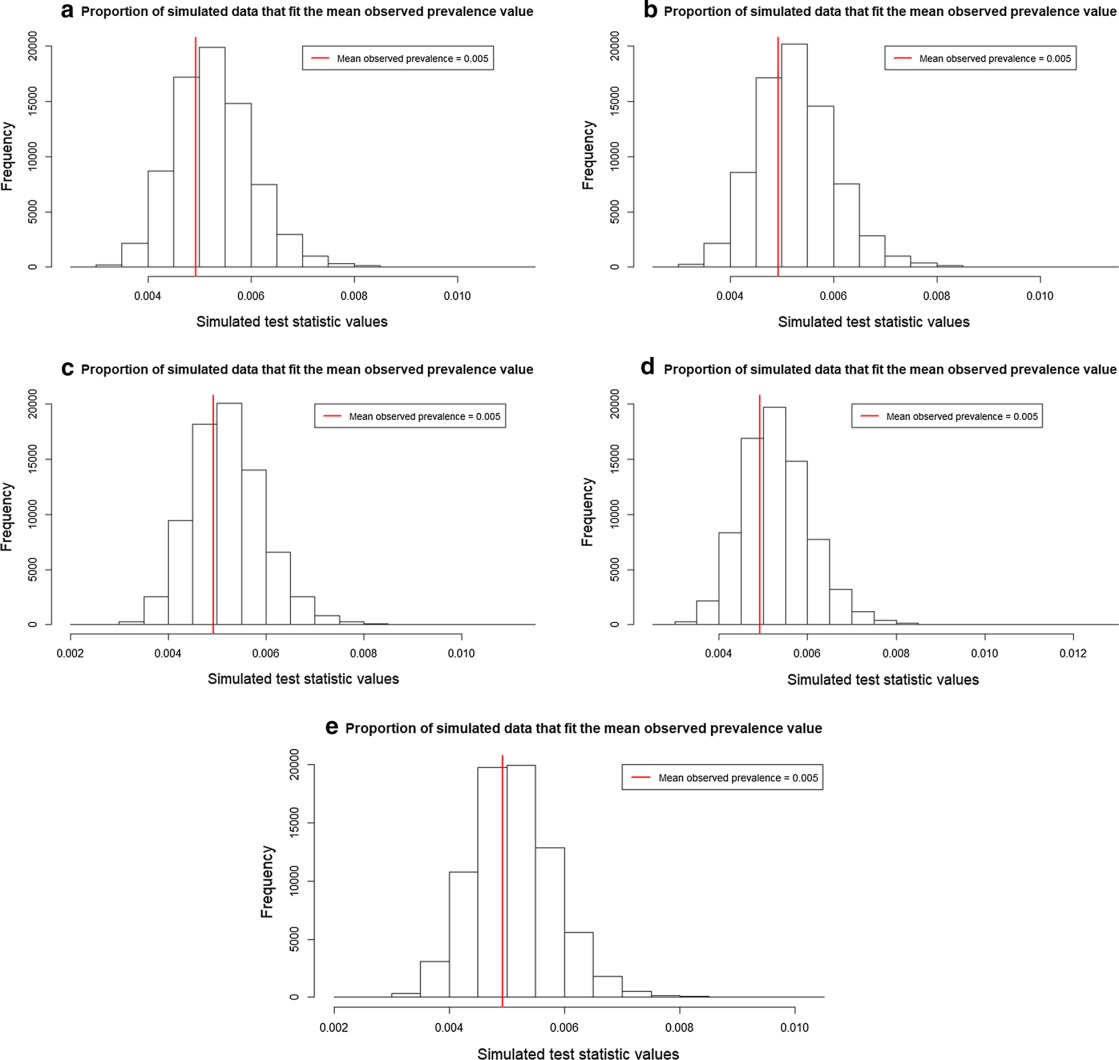
Proportion of simulated prevalence data that fit the observed mean prevalence value. **a** SSA = 30 m. **b** SSA = 90 m. **c** SSA = 250 m. **d** SSA = 500 m. **e** SSA = 1 km. *Abbreviation*: SSA, spatial support of analysis

The maximum and minimum observed prevalence values are 8.5% and 0.33%, respectively. The first validation method shows pp*P*-values for all SSA ranging from 0.64 to 0.67 for the first test statistic (Table 4). This means that simulated data slightly deviate from around 0.14 to 0.17 from the maximum observed prevalence value (Fig. 6). For all SSA it is likely to see a similar number of predicted maximum prevalence values compared to the observed data. For the second test statistic, pp*P*-values ranged from 0.87 to 0.93 (Table 4). This means that simulated data are biased around 0.36 to 0.43 from the minimum observed prevalence data (Fig. 7). For almost all SSA, simulated data predict a higher number of minimum prevalence values compared to the observed data. For the last test statistics, pp*P*-values ranged from 0.59 to 0.67 (Table 4), showing that simulated data deviate from around 0.09 to 0.17 from the mean observed prevalence value (Fig. 8).

**Table 4:** Resulting pp*P*-values for the test statistics: maximum (8.5%), minimum (0.33%) and mean (0.5%) prevalence values at five increasing SSA

Spatial supports of analysis	pp*P*-value (maximum)	pp*P*-value (minimum)	pp*P*-value (mean)
30 m	0.66	0.87	0.66
90 m	0.67	0.86	0.66
250 m	0.66	0.88	0.63
500 m	0.66	0.87	0.67
1 km	0.64	0.93	0.59

*Abbreviation*: pp*P*-value: posterior predictive *P*-value

Results from the second validation method show that all models have a high ability to predict prevalence values, with AUC values of 0.91 for SSA = 30 m, 90 m, 250 m and 500 m, and 0.93 for SSA = 1 km. All models have a good ability to predict the positive number of SCH cases. Models with SSA = 30 m, 90 m, 250 m and 500 m models have AUC values of 0.83, while the 1 km SSA model presents a lower AUC value of 0.79, showing a decrease in the ability to predict the positive number of SCH cases.

## Discussion

Schistosomiasis modelling studies have commonly used environmental risk factors as drivers for disease exposure and transmission [69, 70]. The studies so far have used spatially misaligned environmental variables at different spatial supports of analysis, ignoring MAUP effects on the parameter estimates, predictions, and the relationship between disease morbidity indicators and risk factors. This study is the first effort to quantify the effects of modifying the areal unit (i.e. spatial support) of NDVI, NDWI, LSTD, LSTN, E and NDWB, on model parameter estimates and their uncertainties. Uncertainty may be quantified using measures of accuracy or imprecision [15]. We evaluated uncertainty using measures of imprecision based on the nature of the disease and the survey data available and quantified it using credible intervals in a Bayesian setting. We applied it to *S. japonicum* infection modelling in the Mindanao region, the Philippines.

Our findings show that the environmental risk factors NDVI, NDWI, LSTD, LSTN and E behave similarly when increasing the SSA from 30 m to 1 km (Table 3). An increase in SSA from 30 m to 500 m does not represent any significant changes in parameter estimates. Conversely, for SSA = 1 km, all show a considerable increase in their estimates. The reasons are explained below.

NDVI has a positive effect on SCH, meaning that higher NDVI values increase the prevalence of infection. This is explained by the positive relationship between vegetation, moisture and snail density [37]. NDVI effects are not significant for SSA < 1 km, because NDVI is an indicator of greenness that is mainly effective for arid areas and Mindanao is not arid. However, the NDVI effect becomes significant on the prevalence of SCH infection for SSA = 1 km. This could be because NDVI effects on SCH prevalence are greater at global scales [8] than at local scales. This might be explained by the fact that prevalence values al local scales can vary significantly at nearby locations, as it depends not only on the nature of the parasite life-cycle, which requires optimal habitat conditions (i.e. environmental conditions), but also on sanitation conditions on the area [71]. The increase in uncertainty values with increasing SSA is due to the coarse areal pixels ≥ 250 m resolution that does not reliably represent rice paddy fields. Those are substantially smaller than 25 ha, i.e. are covered by at most four pixels [72].

For SSA = 30 m, 90 m, 250 m and 500 m, LSTD, LSTN and E have a significant negative effect on SCH prevalence. Conversely for SSA = 1 km, their parameter estimates are close to zero. This means that when the areal unit reaches 1 km, the effect of these covariates on the prevalence of SCH infection becomes non-significant. This is also observed from the credible intervals of these covariates for the 1-km SSA model. The reason is that the homogeneity of the covariate values increases when increasing the SSA. LST is a proxy of the ambient temperature of the air, which reflects the thermal conditions of shallow waters [27]. Its negative relationship with the prevalence of infection could be explained by the fact that temperatures above 19–20 °C do not influence the release of cercariae from the infected host to the infection foci [73], as well as temperatures below approximately 15 °C arrest the development of *S. japonicum* in the snail host [74]. The minimum LST value at night is around 21 °C, while the maximum LST value during the day is 31 °C. LSTD and LSTN uncertainty values for SSA = 1 km are remarkably high as compared to other SSA. This is explained by the coarse LSTD and LSTN areal pixels of 1 km^2^ that cannot reliably represent low and high temperature zones in city areas that range from 0.02 to 3 km^2^ [27]. Elevation has a negative effect on SCH. This was expected as in areas with high elevation values (> 2300 m) the risk of infection is low [75]. Conversely, the risk of infection is high for elevation areas below 900 m. Elevation uncertainty values are similar for all SSA, except for SSA = 1 km, where its value considerably decreases to 0.34. Here we see the effect of the gradual changes of elevation in Mindanao region are gradual and without steep slopes [27]. Using data directly at the 1-km SSA could give reliable elevation values, but with a non-significant effect on the disease prevalence.

For NDWB and NDWI, an increase in SSA from 30 m to 250 m represents non-significant changes in parameter estimates, which range from − 1.06 to − 1.02 for NDWI, and from − 0.31 to − 0.28 for NDWB. Conversely, when increasing the SSA to 500 m, parameter estimates change to − 0.8 and − 0.38 for NDWI and NDWB, respectively. For SSA = 500 m and 1 km, NDWI estimates increase, having a less significant effect on SCH prevalence, again due to the increase in the homogeneity of the covariate values when increasing SSA. Higher NDWI values show the presence of potential hidden infection foci. Nevertheless, results show that NDWI presents a negative effect on SCH (Additional file 3: Table S1). This could be because NDWI cannot efficiently suppress the signal from build-up land mixing enhanced water features with build-up land noise. Thus, build-up noise could also have high NDWI values [40]. According to Gu et al. [76] NDWI values lower than 0.3 indicate the presence of drought areas. In our study area, we found that around 77% of Mindanao present drought conditions, explaining the negative effect on the disease. NDWB estimates decrease when increasing SSAs (Table 3), specially for SSA = 500 m and 1 km, but their significance on SCH prevalence increases. A possible explanation is that people that move larger distances to water bodies are most likely to get infected. This could be because at spatial supports < 1 km, NDWB values seem to be more homogenous than at smaller spatial supports, showing a weaker relationship with the disease (Additional file 3: Table S1). For spatial supports > 1 km, neighbouring pixels present more heterogeneous values, which could be because of the aggregation process, but also because of the use of some kind of transportation media that allows apparent reduction of travel distances in a relatively large area (1 km^2^). Clearly, transportation (type of road and media of transportation) plays an important role [77].

Uncertainty values for NDWI decrease when increasing the SSA, with a minimum of 0.44 for SSA = 500 m. Clearly, NDWI data originally available at SSA = 250 m are more reliable than values modified to larger SSAs. Using ordinary kriging for interpolation increases the variance in the estimates in a somewhat unrealistic way since it uses a constant mean [58], while in reality, means are different. Uncertainty values of NDWB, for instance, increase with increasing SSA due to the coarse areal pixel units ≥ 0.25 km^2^. Such a size is insufficient to reliable define nearest distances to water bodies in city areas of 0.02 to 3 km^2^.

Our aim was not to compare the performance of the models as we used the same model structure, number and type of covariates in the five models. Thus the model itself is practically the same for all SSA. Although our aim was not focused on model comparison, the resulting DIC values from Additional file 3: Table S1 suggest the use of spatial support sizes below or equal to 250 m in SCH modelling. This is shown by the low DIC values from 86.67 to 140.5 for SSA ≤ 250 m and high DIC values of 143.7 and 147.5 for the 500 m and 1 km models, respectively (Additional file 3: Table S1).

When modelling prevalence of *S. japonicum* infection in Mindanao, the effect of increasing SSA, or modifying the areal unit of analysis, from 30 m to 500 m, produces a gradual and continuous increase on the parameter estimates and their associated uncertainties. For SSA = 1 km, sudden changes occur in the relationship between the risk factors and the prevalence of the disease. This is shown by the non-significant effect of almost all explanatory variables on *S. japonicum* prevalence. Results suggest that the use of environmental data extracted at SSA = 1 km is not appropriate for the modelling of *S. japonicum* prevalence.

A Bayesian statistical method was used to model the disease along with a convolution regression model, which corrected for pure specification bias on our estimates. This is a relevant contribution to the analysis of uncertainties in this type of spatial epidemiological study. For future studies, new trends in geospatial artificial intelligence (geoAI), that could resolve limitations regarding the MAUP for exposure modelling studies, are emerging to model schistosomiasis [78] as well as other diseases [79]. We particularly identified (i) the use of high-performance computing to handle spatiotemporal big data, and (ii) machine and deep learning algorithms implementation to big data infrastructures to extract relevant disease or environmental information [79, 80]. One example is a data-driven method used to predict particulate matter air pollution (PM_2.5)_ in Los Angeles, CA, USA. Here, machine learning was used on spatial big data, i.e. land use and roads, derived from OpenStreeMap, to predict PM_2.5_ concentrations. When generating relative importance measures for the different risk factors, MAUP effects reduced when applying a random forest model that was trained with the distances between the features and the monitoring PM_2.5_ stations, [81]. The rapid development of geoAI methods, their advantage to deal with big data, and their rapid computational time, makes them an attractive and advantageous tool to tackle limitations with modelling schistosomiasis and other diseases. There is still little work done in this field, but we think it is valuable to further explore geoAI solutions to deal with the MAUP, and perhaps other inherent uncertainties produced in disease modelling and mapping.

Finding MAUP effects on the various environmental risk factors used for modelling *S. japonicum* prevalence, is a step forward to the uncertainty analysis in the schistosomiasis, and possibly other diseases. The present research deals with limitations such as the use of aggregated disease data, due to the lack of geolocated individual-level surveys. It also provides a robust method for the selection of an appropriate spatial data structure, which at the same time, enables the acquisition of more reliable parameter estimates, and defines a clear relationship between the risk factors and the disease. From the public health perspective, this research can support helminth control programmes by providing less uncertain models and maps. Epidemiologists and health scientists could use these maps to identify risk areas for the control and prevention of the disease [12, 82], which in the case of schistosomiasis, is generally based on mass drug administration campaigns addressed to the identified at-risk populations. The provision of reliable information is relevant to guide mass drug administration campaigns by enhancing the assessment of the infection risk, understanding its potential impacts on human health [15, 83] and avoiding erroneous conclusions and decisions about the spatial distribution of schistosomiasis [15, 27]. This research is also relevant to evaluate the effectiveness of mass drug administration campaigns, as it could guide the identification of persistent hot spots, or places where prevalence of infection remains despite mass drug administration efforts [71]. It is known that despite the implementation of mass drug administration campaigns, some places do not show a decrease in local SCH transmission. This is because these campaigns do not only depend on the nature of the parasite life-cycle and the poor sanitation conditions, but also on the local environmental factors, drivers for SCH transmission. Finding relevant environmental factors at local level would allow more intensive efforts at persistent hot spots.

## Conclusions

The present study shows a clear MAUP effect on *S. japonicum* modelling. An increase in parameter estimates and their associated uncertainties occurs when increasing the spatial support of analysis (SSA). It also showed that using environmental data extracted at SSA = 1 km is not relevant for *S. japonicum* prevalence of infection at this specific extent of analysis, as this leads to wrong conclusions about the distribution of the disease and its relationship with the potential risk factors. Thus, the use of maps based upon this information is to be avoided as these may guide health scientists in the control or prevention of the disease astray. The results from this study could guide other disease modelling studies as they suggest a spatial support sizes at which environmental information has no longer a significant effect on the disease, and which data structure is recommended for the modelling. Epidemiologists, decision makers and health scientists could thus benefit from those, e.g. to better understand and quantify MAUP effects on the relationship between the disease and its risk factors, and to provide reliable maps that are useful for disease control and prevention.

## Supplementary information

**Additional file 1: Table S1.** Survey data aggregated at the barangay level. These data show the number of positive cases (y) and the total number of sampled people (*n*) in a barangay (k).

**Additional file 2: Text S1.** Code for the convolution model used in OpenBUGS. It includes the prior distributions used for the covariate and spatial parameters and the model itself. The model uses two indexes, *k* for the barangay and *j* for the number of city pixels within barangays. The betas are the individual-level regression coefficients, *m* is the number of city pixels in a barangay. Covariates are *ndvi*: normalized difference vegetation index, *ndwi*: normalized difference water index, *lstd*: land surface temperature day, *lstn*: land surface temperature night, *e*: elevation, *ndwb*: nearest distance to water bodies. The spatial parameter is represented as *s*.

**Additional file 3: Table S2.** Regression coefficient estimates, variance of spatial random effect, correlation decay coefficient and deviance information criteria for each risk factor at five descending spatial supports of analysis.

**Additional file 4: Figure S1.** Residual plot for the five increasing spatial supports of analysis. **a** SSA = 30 m. **b** SSA = 90 m. **c** SSA = 250 m. **d** SSA = 500 m. **e** SSA = 1 km. The x-axis represents the fitted prevalence values for the five spatial supports of analysis. The y-axis represents the residuals calculated by the difference between the observed and predicted prevalence values.

## Data Availability

Data supporting the conclusions of this article are included within the article and its additional files. The datasets used and/or analysed during the present study are available from the corresponding author upon reasonable request.

## Abbreviations

ASTER: advanced spaceborne thermal emission and reflection radiometer
AUC: area under the curve
BUGS: Bayesian inference using Gibbs sampling
E: elevation
EO: Earth Observation
geoAI: geospatial artificial intelligence
GDEM: global digital elevation map
GIS: geographical information systems
LSTD: land surface temperature day
LSTN: land surface temperature night
MAUP: modifiable areal unit problem
MCMC: Markov chain Monte Carlo
MODIS: moderate resolution imaging spectroradiometer
NDVI: normalized difference vegetation index
NDWB: nearest distance to water bodies
NDWI: normalized difference water index
ROC: receiver operator characteristic
SCH: schistosomiasis
SSA: spatial support of analysis
USGS: United States Geological Survey
