# Supplementary figures and images for: Modelling the impact of MAUP on environmental drivers for Schistosoma japonicum prevalence
Source: Parasit Vectors. 2020 Mar 2;13:112. doi: 10.1186/s13071-020-3987-5 (PMC7053105; doi:10.1186/s13071-020-3987-5)

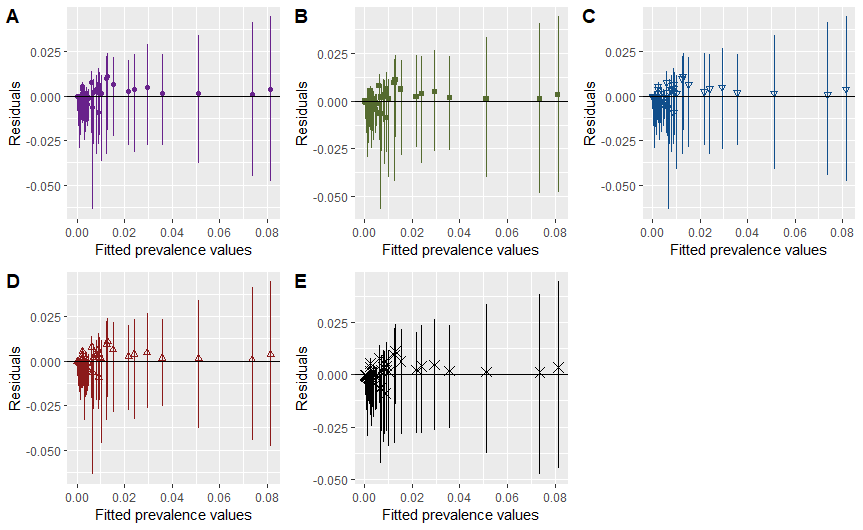

Supplement: Supplementary file 4 — Additional file 4: Figure S1. Residual plot for the five increasing spatial supports of analysis. a SSA = 30 m. b SSA = 90 m. c SSA = 250 m. d SSA = 500 m. e SSA = 1 km. The x-axis represents the fitted prevalence values for the five spatial supports of analysis. The y-axis represents the residuals calculated by the difference between the observed and predicted prevalence values. [file 13071_2020_3987_MOESM4_ESM.tiff]
